# Ancient DNA Reveals the Earliest Evidence of Sheep Flocks During the Late Fourth and Third Millennia BC in Southern Iberia

**DOI:** 10.3390/ani14243693

**Published:** 2024-12-20

**Authors:** Gabriel Anaya, Juan Manuel Garrido, José Antonio Riquelme, Rafael Mª. Martínez, Alberto Membrillo, José Antonio Caro, Ana Pajuelo, Adrián Ruiz, José C. Martín de la Cruz, Antonio Molina

**Affiliations:** 1 MERAGEM (AGR-158) Research Group, Department of Genetics, University of Córdoba, CN IV KM 396, 14014 Cordoba, Spain; b22ancag@uco.es; 2 INREPA (HUM-262) Research Group, Department of History, University of Córdoba, Plaza Cardenal Salazar, 3, 14003 Cordoba, Spain; z02gaanj@uco.es (J.M.G.); jriquelme@uco.es (J.A.R.); l62ruexa@uco.es (A.R.); ch1macrj@uco.es (J.C.M.d.l.C.); 3 Department of History, University of Córdoba, Plaza Cardenal Salazar, 3, 14003 Cordoba, Spain; l82masar@uco.es; 4 Department of Specific Didactics, University of Córdoba, Avda. San Alberto Magno s/n, 14004 Cordoba, Spain; b72depoa@uco.es; 5 Cuaternario y Geomorfología (RMN-273) Research Group, Department of History, University of Córdoba, Plaza Cardenal Salazar, 3, 14003 Cordoba, Spain; jacaro@uco.es; 6 TELLUS (HUM-949) Research Group, Department of Prehistory and Archaeology, University of Seville, Calle San Fernando 4, 41004 Sevilla, Spain; appando@us.es

**Keywords:** late fourth and third millennia BC, livestock production, sheep, genomics, ancient DNA, sequencing

## Abstract

The Spanish Merino is the most significant sheep breed globally due to its economic and cultural importance in human history. It has also had a substantial influence on the development of other Merino and Merino-derived breeds. Historical sources indicate that crossbreeding to produce finer, higher-quality wool was already taking place in the south of the Iberian Peninsula during the Roman era. This evidence suggests that individuals with a racial pattern very similar to that of the modern Merino may have already existed on the peninsula. The presence of the skeletal remains of these animals at various human settlements dated to the late fourth and third millennia BC could provide insights into the genomics of these ancestral sheep. This study analyses ancient DNA extracted from nine skeletal remains from three archaeological sites in Southern Iberia, dated to the third millennium BC. The samples were sequenced and aligned with the ovine genome. The genetic distances observed among the samples indicate a closer relationship between several animals from the Marinaleda (Seville) and Grañena Baja (Jaén) sites. The study of the slaughter/death age profiles identified at La Minilla (La Rambla, Córdoba) suggests an approach centred on meat exploitation, while the data from Marinaleda (Seville) and Grañena Baja (Jaén) indicate the potential exploitation of secondary resources. A review of the composition of these small ruminant herds could provide insights into the type of secondary resource exploitation that may have been prioritised. Our aim is to investigate the presence of distinct production systems, differentiating between those aimed primarily at meat use and those focused on secondary products. This is the first approach to exploring the genetic evidence for sheep livestock related to its productive use during this period and in this geographical area.

## Simple Summary

The Spanish Merino is one of the most significant sheep breeds globally, not only because of the exceptional quality of its wool but also due to its considerable economic and historical impact. Historical sources indicate that crossbreeding to produce finer, higher-quality wool was already taking place in the south of the Iberian Peninsula during the Roman era. This evidence suggests that individuals with a racial pattern very similar to that of the modern Merino may have already existed on the peninsula. A review of small ruminant herd composition and death/slaughter patterns could provide insight into the type of secondary resource exploitation that may have been prioritised. In the present study, we explore this possibility by examining genomic evidence from the late fourth and third millennia BCE in Southern Iberia. Our aim is to investigate the presence of distinct production systems, differentiating between those aimed primarily at meat use and those focused on secondary products.

## 1. Introduction

The Spanish Merino is one of the most significant sheep breeds globally, not only because of the exceptional quality of its wool but also due to its considerable economic and historical impact. Indeed, the Merino breed has played a crucial role in the development of most Merino and Merino-derived breeds worldwide. While the precise origins of the current Merino breed remain uncertain, Roman historical documents suggest that the Iberian sheep serves as its ancestral genetic base [1]. According to these records, Roman livestock practises involved crossing the dark-wool Iberian sheep (which had very fine wool) with foreign white-wool sheep brought from the Atlas Mountains in North Africa to obtain animals with the finest wool fibre and white vellon [2]. This evidence demonstrates that, as early as Roman times, some sheep flocks were being specialised for secondary products such as wool. The remarkable fineness of the fibre produced by these endemic Iberian animals raises intriguing questions about whether differentiated systems of use existed even earlier than this period. In the present study, we explore this possibility by examining evidence from the late fourth and third millennia BCE in Southern Iberia.

To address this, we must confront various challenges. On the one hand, not all the sites have a series of reliable chronologies, and the faunal assemblages studied are often grouped into very broad time periods, further complicating a scenario in which synthetic studies are scarce [3,4,5]. On the other hand, in addition to the evident scarcity of specific published studies on the mammal fauna of certain sites in Southern Iberia, we must also consider the limitations of the assemblages studied, which typically do not exceed a hundred remains. In the case of small ruminant livestock, commonly referred to as ovicaprids (belonging to the subfamily Caprinae), the taxonomic challenge of distinguishing between sheep and goats often leads some studies to report a combined result for the minimum number of individuals (MNIs), which is classified under ovicaprids or caprines.

**Figure 1:**
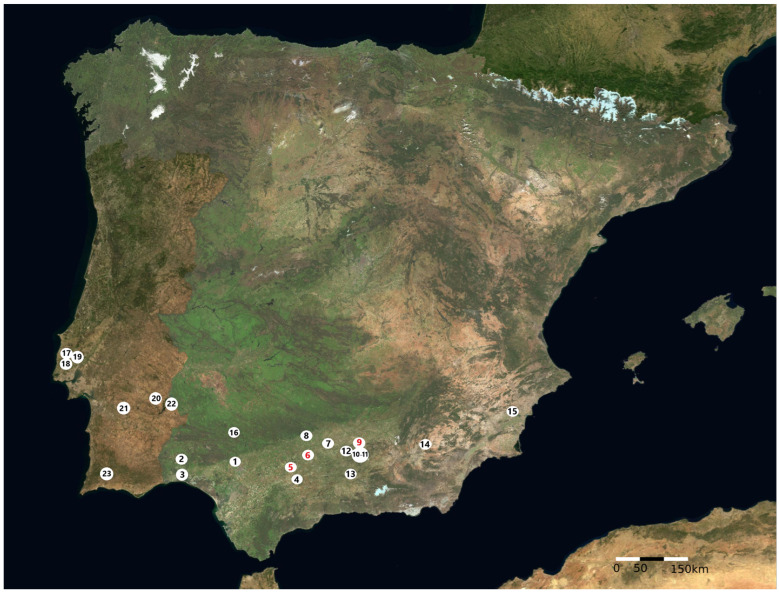
The sites from the late fourth and third millennia BC in Southern Iberia with a minimum number of *Ovis aries* individuals identified. Spain: 1. Valencina de la Concepción (Sevilla), 2. Cabezo Juré (Alosno, Huelva), 3. Papa Uvas (Aljaraque, Huelva), 4. Gilena (Sevilla), 5. Marinaleda (Sevilla), 6. La Minilla (La Rambla, Córdoba), 7. Torreparedones (Baena, Córdoba), 8. Antigua Iglesia de Alcolea (Córdoba), 9. Grañena Baja (Jaén), 10. IA Corte Inglés (Jaén), 11. Ciudad de la Justicia (Jaén), 12. Polideportivo de Martos (Martos, Jaén), 13. Los Castillejos (Montefrío, Granada), 14. Cerro de la Virgen (Galera, Granada), 15. Les Moreres (Crevillent, Alicante). 16. Cueva de los Covachos (Almadén de la Plata, Sevilla). Portugal: 17. Zambujal (Torres Vedra), 18. Penedo do Lexim (Mafra), 19. Ota (Alenquer), 20. Perdigões (Reguengos de Monsaraz), 21. Monte da Tumba (Torrão), 22. Mercador (Mourão), 23. Alcalar (Portimão). The red numbers indicate the sites where the samples were collected for palaeogenomic analysis. Reference map “https://www.ign.es/iberpix/visor/ (accessed on 10 December 2024)”. Iberpix is a cartographic viewer published by the National Centre for Geographic Information (CNIG) and the National Geographic Institute of Spain (IGN) used for the consultation and visualisation of maps and layers of geographic information.

Despite these limitations, based on the available data, and applying an ecological approach, this study aims to provide a general overview using chronological filters and focusing on Copper Age archaeological sites where faunal assemblages were analysed and both the bone remains of *Ovis aries* and a minimum number of individuals (>1) of this species were identified (Figure 1). Subsequently, utilising studies conducted on faunal assemblages excavated at the archaeological sites of Grañena Baja (Jaén), Marinaleda (Seville), and La Minilla (La Rambla, Córdoba) [6,7,8], we will focus on the genetic analysis of nine *Ovis aries* individuals.

The importance of understanding the dynamics of sheep breeding at the end of the fourth and third millennia BCE in the Southern Iberian Peninsula is essential for understanding the transition toward more complex agricultural and pastoral economies. Sheep provided not only meat but also secondary products [9] thus diversifying diet and the available resources. Our aim is to investigate the presence of different production systems, differentiating between those aimed primarily at meat use and those focused on secondary products. This is the first approach to explore the genetic evidence for sheep livestock related to its productive use during the late fourth and third millennia BC in the Guadalquivir Depression.

### 1.1. The State of the Art

The analysis of the populations that inhabited the Southern Iberian Peninsula during the late 4th and 3rd millennia BCE has sparked intense debate regarding social stratification and inequalities. One interpretative approach posits that Chalcolithic social formations functioned as tributary states, where central settlements, inhabited by elites, exerted an exploitative control over the agricultural and metallurgical production of dependent peripheral communities [10,11,12,13,14,15,16,17]. On the other hand, an opposing perspective argues that these societies did not develop institutionalised forms of power or marked social inequalities. Instead, they were organised under communal economic principles, with collective access to means of production and goods, and maintained undifferentiated funerary ideologies. Therefore, these communities would be better described as pre-state societies [18,19,20,21,22,23].

It was suggested some time ago that the predictive strategies of cereal storage and livestock stabling helped the Neolithic and Chalcolithic communities of Southeastern Iberia to achieve an excess production model [12]. In those Copper Age societies of Southern Iberia, where it is believed that an incipient hierarchization and leadership already existed, it was proposed that the appearance of anthropomorphic figures may correspond to community leaders [24], some of whom might have been holding a symbol of power in their hands.

The presence of objects made from exotic raw materials (beads made from ostrich eggshell, ivory, rock crystal, or amber) found in the tombs of the Millares settlement seems to indicate the existence of an incipient elite during the 4th and 3rd millennia BCE [25,26,27]. The same is true for Valencina de la Concepción, where gold, amber, an ostrich egg, and an elephant tusk were found [28,29]. All of this suggests that there were ideological and economic connections between these societies or that they were part of the same networks for the distribution of exotic objects.

Not all of the 3rd millennium BCE sites addressed in our study are the same size. There are large settlements, such as Valencina de la Concepción, with a total area of 468.8 hectares, and a settlement of 235.6 hectares [30].

Medium-sized settlements are evidenced by the excavations at IA Corte Inglés (Jaén) and Ciudad de la Justicia (Jaén) within the Marroquies Bajos site (Jaén), where the layout of the outermost enclosure, or Ditch 5, was estimated to cover an area of at least 113 hectares, of which 34 hectares correspond to the area strictly considered as the settlement [31,32,33]. In the case of Zambujal (Torres Vedras), the area occupied by the Copper Age settlement and the possible necropolis spans approximately 46 hectares [34]. The settlement of the third millennium at Alcalar covers about 20 hectares [35], while the Perdigões archaeological complex occupies about 16 hectares (Márquez et al., 2011). Smaller settlements include Les Moreres (book citation), Penedo do Lexim (Mafra) [36], Ota (Alenquer) [37], Los Castillejos (Montefrío, Granada) [38], and Mercador (Mourão) [39]. The size of the remaining sites cannot be estimated, as they have only been excavated partially.

On reviewing the published data related to faunal lists in which *Ovis aries* remains from the late fourth and third millennia BC in Southern Iberia were identified [6,8,35,38,40,41,42,43,44,45,46,47,48,49,50,51,52,53,54,55,56], a recurrent observation is the predominance of domestic fauna, with the caprine–bovine–porcine triad standing out [57].

The current number of Late Neolithic–Copper Age archaeological sites where faunal remains were studied and a minimum number of *Ovis aries* individuals were identified is shown in Table 1.

Several faunal studies were conducted on the archaeological site of Valencina de la Concepción (Seville), the most comprehensive of which was carried out by Hain (1982), who found that the small ruminant stock (sheep and goat) was the second most exploited. A subsequent study with a smaller sample determined that sheep and goat exploitation ranked third in terms of NRs (number of remains), although it was the second most represented in terms of MNIs [47]. In the latest study, the sheep and goat herds were found to be the second most represented [47]. In all the studies, sheep were more frequently represented than goats. In the Sierra Norte of Seville, the Cueva de los Covachos provides intriguing insights into livestock practises during the transition from the 4th to the 3rd millennium BC. The predominance of caprine bones discovered suggests their primary role in the economy of that society, with only sheep identified at the site [62].

The faunal study results from the Cabezo Juré site (Alosno, Huelva) confirm that sheep and goats represent the second most prevalent livestock group, both in terms of NISP and MNIs [4]. At Papa Uvas, sheep/goat livestock is the most represented along with bovids, with sheep being the most represented species in the first group [56,58,59]. In the archaeological zone of Marroquíes Bajos (Jaén) at the IA Ciudad de la Justicia site, the faunal study showed that sheep and goat husbandry ranked third [64], with sheep outnumbering goats. However, at the Late Neolithic site of Polideportivo de Martos (Martos, Jaén), sheep/goat livestock predominated over bovine and porcine, with the minimum number of individuals (MNIs) for goats twice that of sheep [60,61].

In the Copper Age sequence at Los Castillejos de Montefrío (Montefrío, Granada), small ruminant livestock (sheep and goats) predominated in both the early and late phases; however, during the intermediate period, porcine livestock assumed a primary role [38]. Throughout much of the third millennium BC, goats dominated the small ruminant herds; however, by the end of this millennium, sheep became predominant.

In Phase I of Cerro de la Virgen (Galera, Granada), in the domestic huts, sheep husbandry held the primary position, with more sheep remains identified than goat remains [41] (Table 1). The maximum lengths of six complete metatarsals recovered indicate that they belong to six different individuals [41].

At the site of Les Moreres (Crevillent, Alicante), during the construction phase of Moreres III, sheep and goat husbandry ranked second, becoming the predominant livestock in Moreres IV, where sheep were the only species identified in both phases [55].

One of the largest Copper Age settlements in Southern Iberia is Zambujal (Torres Vedras, Portugal), one of the most extensive faunal studies, with a significant number of identified remains, was conducted [40]. Sheep and goats were the primary focus of livestock exploitation, although there is no significant difference in the number of remains compared to pig remains [40], with sheep more numerous than goats [40]. The faunal study of Penedo do Lexim (Mafra) shows that small ruminants occupied an almost equal position to suids, with the sheep again outnumbering the goats [52].

The archaeozoological record from Ota (Alenquer, Portugal) indicates that the sheep and goat herds constituted the primary source of exploitation, followed by bovines, with more sheep than goats identified [53].

In the sample recovered from Perdigões (Reguengos de Monsaraz, Portugal), sheep and goat husbandry was the second most represented domestic group after suids, although it did not constitute the second highest dietary contribution due to deer hunting. Within the sheep and goat herds, the sheep once again outnumbered the goats [48,54]. A similar situation was found at the Chalcolithic settlement of Monte da Tumba (Torrão, Portugal), where ovine and caprine husbandry ranked second in terms of livestock exploitation, with deer hunting also making a considerable contribution, with significantly more sheep than goats [43]. The same was true at Mercador (Mourão, Portugal), where ovine and caprine livestock ranked second, far behind swine exploitation, with a significant contribution from deer and rabbit hunting [51].

Several other sites, though with a much smaller number of identified remains, can help complete the overall picture. At the Iglesia Antigua de Alcolea site (Córdoba), the primary livestock was pigs, with caprines (sheep and goats) occupying second place. Among the caprines, more sheep than goats were identified [57]. In Gilena (Seville), the sheep and goat herds were the most represented, with sheep being the most represented species within this group [44]. At the Torreparedones site (Baena), ovine and caprine husbandry ranked third, with more sheep than goats [50]. In Phase B of IAP El Corte Inglés, ovine and caprine livestock ranked second, with sheep again outnumbering goats [45]. However, at the Alcalar site (Portimão, Portugal), there was a parity between sheep and goats, with the sheep and goat herds being the second most exploited, after swine farming [63].

In this panoramic view of the late fourth and third millennia BC in Southern Iberia, some settlements that undoubtedly had significant territorial influence are notably absent, such as the case of Los Millares. Around 20,000 bone remains from domestic livestock were studied there, and we know that ovine and caprine husbandry held a priority position, with more sheep identified than goats. However, we do not yet have an estimate of the minimum number of individuals [65]. In the faunal study of Vila Nova de São Pedro (Azambuja, Portugal), no distinction was made between *Ovis* and *Capra* [66]. These are just a few of the most significant late fourth and third millennia BC settlements that will likely provide interesting results soon.

Currently, the general impression is that the prevalence of suids at Copper Age sites in Southern Iberia depends more on the type of context than on their actual role. This is likely to be related to feasting and communal consumption [67], with caprines actually being the primary livestock. In any case, the number of suids appears to have increased proportionally at sites in Southwestern Iberia [57].

### 1.2. Geographical Area

The bone remains used for the genomic study of sheep farming in the Guadalquivir Valley were collected from Copper Age archaeological contexts at the sites of Grañena Baja (Jaén), Marinaleda (Seville), and La Minilla (La Rambla, Córdoba) (Figure 1).

The archaeological site of Grañena Baja is located 9 km north of the city of Jaén, on the left bank of the Guadalbullón River. Phase III is defined by the discovery of 108 structures of various types, including ditches, habitation sites, storage facilities, combustion areas, funerary structures, etc. Among these, a considerable number of faunal remains were found [68]. Samples of *Ovis aries* were recovered from Phase III, contexts NE 25 and NE 131 [6], dating to a period close to the transition from the 4th to the 3rd millennium Cal BC. The two individuals taxonomically identified as sheep (over two years old) were genomically analysed.

As a result of the casual discovery of a series of bell beaker vessels in the surrounding area (1986) and with plans for new construction (1989), an emergency archaeological excavation was conducted at the La Minilla site (La Rambla, Córdoba). The faunal remains come from the pit structures, with both structures aligned parallel to each other, which, given the chronology, likely represent an enclosure that would have encompassed a habitation area roughly coinciding with the present-day village [69]. The faunal study indicates that of the domestic fauna, goats were the second most represented, with a greater number of sheep remains identified than goat remains. The study on the age at slaughter/death of the nine sheep individuals revealed that seven had died or were slaughtered before 24 months of age, while two surpassed this age [8]. The two samples of *Ovis aries* were recovered from Trench 1 (Z1) and can be dated to 2835–2460 BC.

The Marinaleda site (Seville) is situated near the eponymous locality, and the fauna remains recovered originate from an archaeological excavation conducted prior to the construction of a school. In the highest area of the hill occupied by the site, Sector C was excavated, which proved to be the most interesting due to its significant concentration of structures and material remains. Given the urgent nature of the intervention, it was necessary to select certain structures for excavation, including a large six metre wide ditch (Ditch 2), a wall complex adjacent to this ditch, and finally, a trilobulated underground structure [7].

The fill sediment of the three entry structures was very homogeneous, clayey, and dark with some stone blocks, allowing for the differentiation of various stratigraphic units. In all cases, the walls sloped until they ultimately converged, forming an impressive underground space with three interconnected compartments reaching a maximum depth of 2.10 m. Among the materials recovered from the stratigraphy (UE82, UE73, UE40, UE44, UE30) of this trilobate structure are the samples of the *Ovis aries* presented in this work, one of which was subjected to radiocarbon dating (C14 AMS) and dated to two chronological intervals ranging from 2346 to 2138 Cal BC (Table 2). This chronology aligns with the archaeological materials recovered throughout the sequence from which bell beaker vessels with impressed decoration and highly burnished black ceramics stand out, accompanied by a wider variety of undecorated elements. Although no comprehensive study of the fauna has yet been published, it was decided that the five bone remains taxonomically identified as sheep and genomically analysed in this study belonged to adults.

## 2. Materials and Methods

### 2.1. Archaeological Approach

For the archaeological sites of Grañena Baja and La Minilla, taxonomic identification was conducted using the comparative collection, along with assemblages housed at the Archaeobiology Laboratory (ArqBio)-CSIC and the Instituto de Arqueociências in Lisbon, facilitated by research stays undertaken while the material was being studied. In the case of Marinaleda (Seville), anatomical and taxonomic identification was conducted using reference collections from the Faculty of Veterinary Medicine and the Prehistory Laboratory at the University of Córdoba, along with specific atlases and studies on agrarian methods and domestic customs [38,71,72,73]. The criteria established for distinguishing between sheep and goats, as outlined in recent studies [74,75,76], were applied alongside direct osteological comparisons. Additionally, loose teeth were included in the overall count and in the calculation of the MNIs. Age determination was carried out following the criteria established by Silver [77] regarding dental eruption and wear, as well as using epiphyseal fusion and cranial suture fusion.

For sampling, stratigraphically related and chronologically dated bone remains, identified as sheep from different archaeological contexts, were selected. Extraction was performed on compact areas of the bone, avoiding fractures, perforations, or any features that could have exposed the osteocytes and led to DNA degradation (Table 3).

### 2.2. DNA Purification

The 9 bone remains were processed in the Ancient DNA Laboratory of the MERAGEM research group (University of Cordoba, Cordoba, Spain), where the aDNA was purified following the Yang protocol [78]. Samples from 3 autochthonous Spanish breeds from the south of the Peninsula were employed for comparison with the aDNA. Two Spanish Merino (ME) were selected for their wool and/or wool–meat use, two Black Merino (MN) were selected due to their ancestral origin, and two individuals from the Segureña breed were analysed for their high meat use. Blood from the 6 individuals was collected by jugular venipuncture in vacutainers, with EDTA K3 as the anticoagulant. The genomic DNA was purified with the DNeasy Blood and Tissue Kit (QIAGEN, Hilden, Germany), following the manufacturer’s instructions.

### 2.3. Sequencing, Alignment, and Quality Control

The aDNA from the 9 bone remains and the DNA from the 6 modern sheep were sent to the National Centre of Genomic Assays (CNAG) (Barcelona, Spain). Paired-end sequencing libraries were constructed according to the manufacturer’s instructions (Illumina Inc., San Diego, CA, USA), and then analysed for size distribution using TapeStation, quantified using Qubit, and sequenced on the Illumina NovaSeq 6000 platform (Illumina Inc., San Diego, California, USA) at PE150. A total of 222.7 GB of data was generated. The quality control of the raw data was conducted with fastqc V0.11.9 software (https://www.bioinformatics.babraham.ac.uk/projects/fastqc/ accessed on 24 August 2024). After filtering, the adapters were removed using fastp v0.23.4 software [79]. The remaining high-quality sequences were aligned with the GCA_016772045.1_ARS-UI_Ramb_v2.0 sheep genome (previously indexed), using the Burrows–Wheeler Aligner (BWA) v0.7.18 software with the “-men” command [80]. Next, the bam files generated for each sample were sorted using the “sort” command in Samtools v1.12 [81]. Duplicates were marked and removed using the “MarkDuplicates” and “RemoveDuplicates” commands in Picard v3.1 software (Picard Toolkit. Cambridge, UK, 2018). The samples were then filtered to keep only the reads with paired reads mapped with quality values of 20, and an alignment score above 100, using Samtools v1.12 software [81].

### 2.4. Variant Calling

The genotype likelihood was calculated by adding the allelic depth, genotype depth, and strand bias, using the *Ovis aries* reference genome GCA_016772045.1_ARS-UI_Ramb_v2.0 to generate a bcf file using bcftools (https://github.com/samtools/bcftools, accessed on 24 March 2023). Finally, the genotypes were called with bcftools to generate the vcf file, which was converted to a binary file using plink v1.9 software [82].

### 2.5. Genomic Comparison Between Ancient Samples

In order to compare the ancient samples, Nei’s genetic distance [83] was calculated with the StAMPP-1.6.3 R package [84] and visualised in a tree using the ape 5.5 package [85] and the Unweighted Pair Group Method with the Arithmetic mean (UPGMA). Samples were sexed using the plinkv1.9 software [82], and inbreeding coefficient (F) and multilocus heterozygosity were estimated with the “calcdiversity” function of R package Sambar [86]. Thereafter, Nei’s genetic distance was used for the principal coordinate analyses (PCoAs) performed using the function “pcoa” of the R package ape-5.7.1 [85].

### 2.6. Genomic Homology with Modern Breeds

In order to analyse the complete sequence of the aDNA samples and thereby obtain the highest possible level of information, the remains were individually compared with modern breeds to check similarities through a Principal Components Assay (PCA) using PLINK v1.9 software [82].

## 3. Results

### 3.1. Genomic Comparison Between Samples from Different Sites

**Figure 2:**
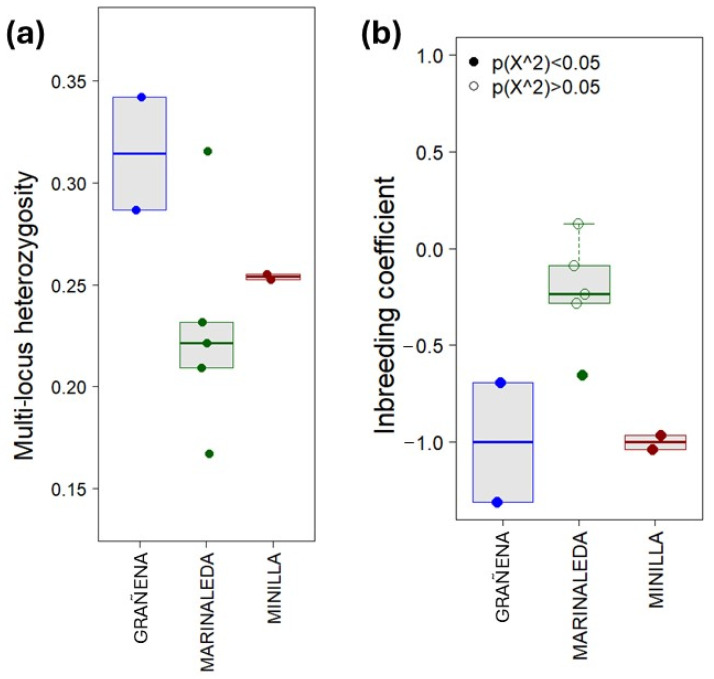
Genomic diversity of bone remains by site. (**a**) Multi-locus heterozygosity at individual level. (**b**) Inbreeding coefficient at individual level.

The nine samples were sexed as females. Figure 2 shows the individual genomic diversity as multi-locus heterozygosity (Figure 2a) and inbreeding coefficient (Figure 2b). Samples from Grañena showed the highest mean levels of multi-locus heterozygosity, while Marinaleda showed the lowest. The dispersion of the values was highly significant in the Marinaleda remains.

**Figure 3:**
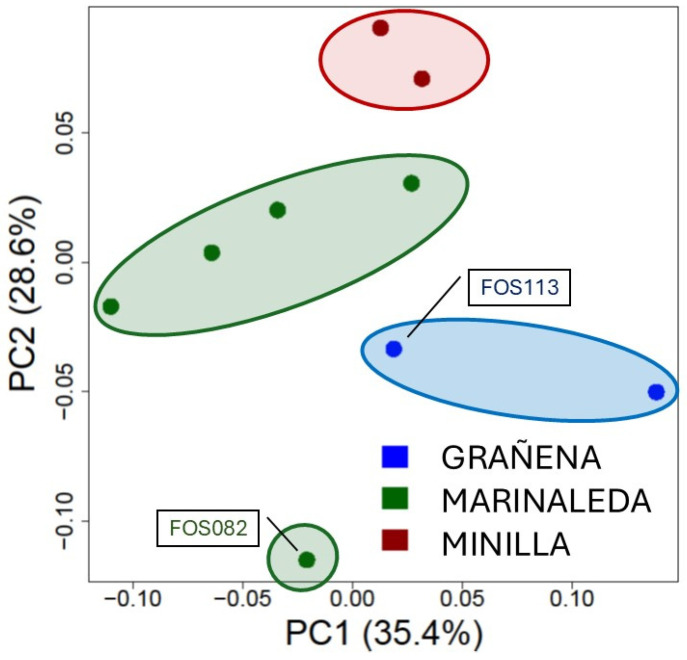
The Principal Coordinates Analysis of the bone remains from the three sites of the study. The red ellipse corresponds to the Minilla remains, the green ellipse corresponds to the Marinaleda bones, and the blue ellipse corresponds to the samples from Grañena.

The Principal Coordinates Analyses (PCoA) calculated for the ancient samples showed how the sheep cluster was in the same position in the different sites, although one sample from Marinaleda (FOS082) was located out of the cloud formed by the rest of the samples from this site and nearer to the Grañena samples. The first component captured 35.4% of the variability, while the second PC axis accounted for 28.6% of the total variance (Figure 3).

**Figure 4:**
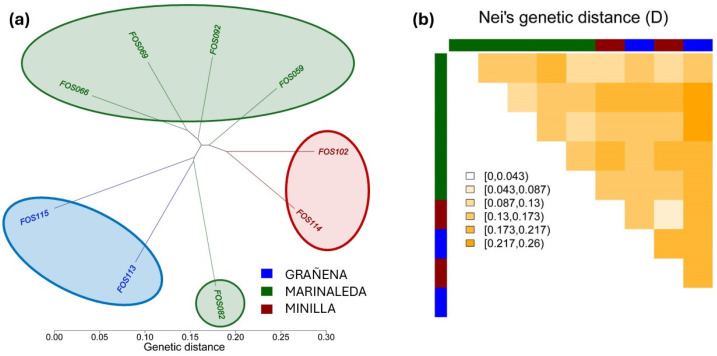
(**a**) Dendrogram depicting Euclidean genetic distances between individuals. (**b**) Nei’s Genetics distances between individuals. Red ellipse corresponds to Minilla remains, green ellipse to Marinaleda bones, and blue ellipse to samples from Grañena.

The dendrogram between individuals indicates a high level of similarity between animals from the same location that are in the same branch. However, sample FOS082 belonging to the Marinaleda site is included in the branch of samples belonging to the Grañena site (Figure 4a). Overall, the genetic distances did not show major differences between the different sites (Figure 4b).

**Figure 5:**
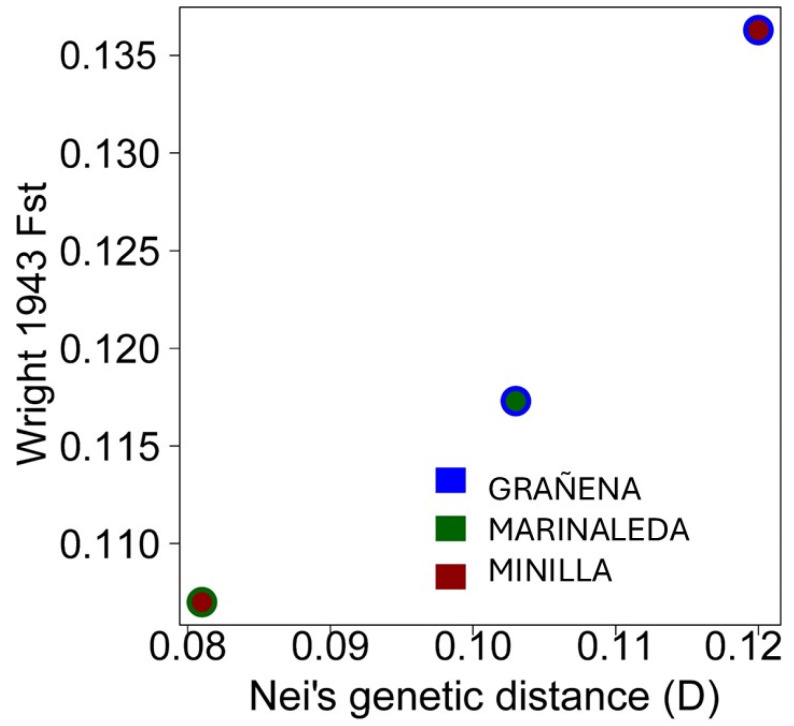
Representation of genetic distances of Wright 1943 Fst and Nei (D), between sites of Grañena, Marinaleda, and Minilla.

A comparison of the genetic distances of Fst from Wright 1943 and Nei (D) are shown in Figure 5. Both genetic distances show the same tendency, with the most similar sites being the Marinaleda and La Minilla sites, while the most genetically different animals are those belonging to the Minilla and Grañena sites.

### 3.2. Genomic Comparison Between Ancient Remains and Modern Breeds

**Figure 6:**
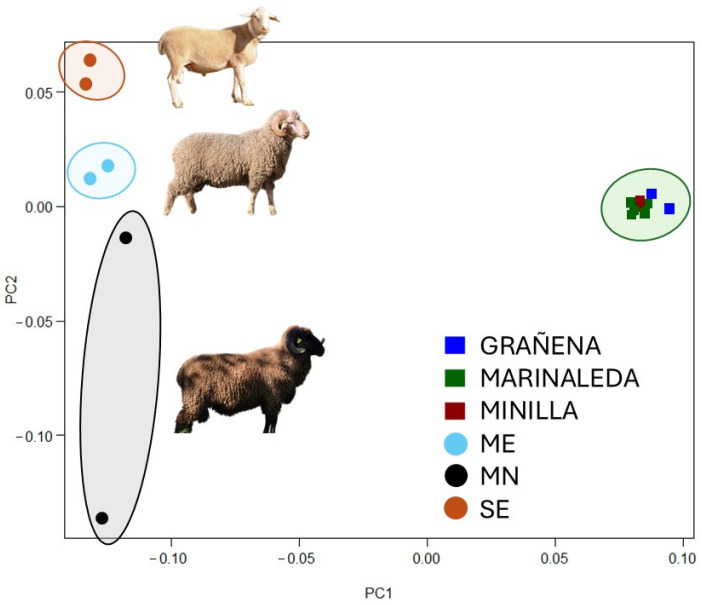
The Principal Component Analysis of the bone remains from the three sites in the study compared to the modern Segureña (SE), Merino (ME), and Black Merino (MN) breeds.

The comparison between the ancient samples and modern breeds was conducted both jointly, to provide an overall view, and individually, to maximise the number of available DNA markers for each bone remain. When the aDNA were compared to the modern DNA samples, it was observed that all the bone remains clustered in a cloud, regardless of their site, were distinct from the modern sheep, which, in turn, were also clustered according to their breed (Figure 6).

**Figure 7:**
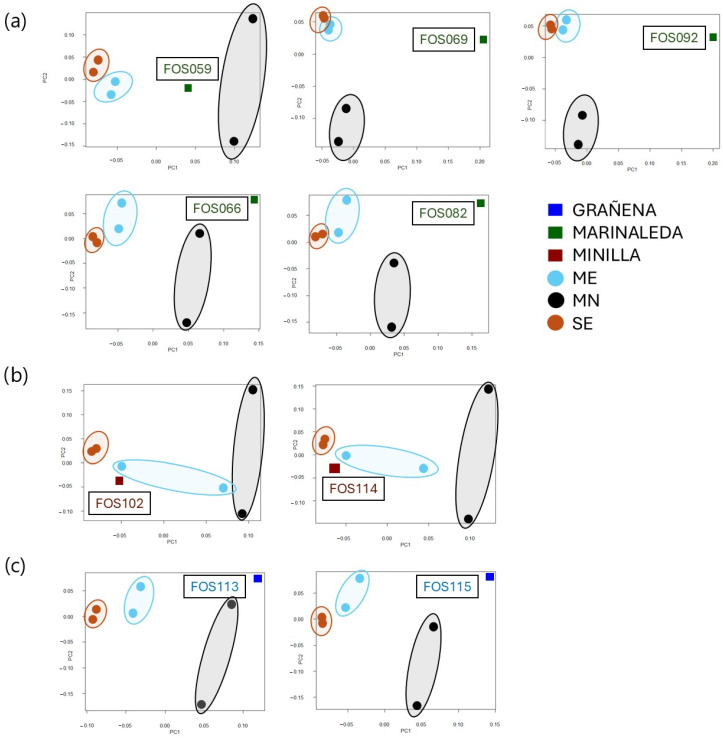
The individualised Principal Component Analyses of the bone remains from the Marinaleda (**a**), La Minilla (**b**), and Grañena (**c**) sites of the study compared to the modern Segureña, Merino, and Black Merino breeds.

The individualised analyses indicated that FOS69 and FOS92 samples from the Marinaleda site exhibited significant genetic distance and equidistance from modern breeds, particularly when compared to the Merino and Black Merino. On the other hand, samples FOS66, FOS82, FOS113, and FOS115 from the Marinaleda and Grañena Baja sites displayed a significant genetic divergence from both the White Merino and the Segureña sheep, yet clustered more closely with the Black Merino. Finally, the samples from La Minilla demonstrated a greater genetic affinity with one White Merino individual and the Segureña sheep (Figure 7).

## 4. Discussion

So far, based on the faunal records of Late Neolithic–Copper Age settlements in Southern Iberia, it is clear that sheep and goat husbandry held a prominent position alongside pig farming. Pig remains are often found in large quantities, which can be interpreted as evidence of collective consumption events [67]. Domestic caprines were the best-adapted animals to the environment and held a predominant economic role in the region from the Neolithic to the Roman periods. On the other hand, pig remains are usually easier to identify due to their distinct anatomical features, whereas many domestic caprine remains are classified as indeterminate mesomammals, leading to their underrepresentation [6].

In general terms, without delving into the environmental and geographical characteristics of each habitat, it seems that the primary meat source for these human communities was provided by pigs and supplemented by ovicaprid and bovine sacrifices.

It is highly probable that during this period, sheep and goat herds were managed together. Recent studies have shown that mixed herds enhance efficient resource utilisation in pasture systems. This synergy arises from the complementary feeding habits of the two species: sheep primarily graze on low-lying grasses, while goats exhibit a preference for browsing on shrubs and higher vegetation. This grazing behaviour not only reduces competition for food but also promotes a more extensive growth of ground-level grasses, as goats consume shrubs and reduce vegetative overgrowth, which frees space for grass growth [87]. Goats exhibit more exploratory and aggressive behaviour than sheep, and due to their vocalisations and high state of alertness, can serve as effective sentinels, potentially warning the herd of the presence of a predator. Additionally, in extensive grazing systems, the role of an experienced, older goat often proves valuable in guiding movements of the herd thus facilitating group cohesion [88]. All these reasons suggest that sheep and goats could have been managed together in single, mixed herds during the late fourth and third millennia BC. Recent advances in geoarchaeological studies of stabling floors could shed light on this issue; however, for now, the ethnoarchaeological results are promising [89].

Regarding the different sites, based on the number of identified bones remains and the minimum number of individuals, in most cases, sheep had higher prevalence than goats. A study on aDNA and the application of ZooMS analysis suggest a possible overrepresentation of goats when using the osteomorphological criteria of Zeder and Pilaar (2010), which are based on mandibular teeth to distinguish between sheep and goats [90]. In any case, even when accounting for a possible overestimation of goat remains compared to sheep, herds contained a majority of sheep over goats during the late fourth and third millennia BC. This preference may be attributed to the generally docile nature of sheep, which likely facilitated their management and grazing especially in the Guadalquivir Basin, which is rich in pastures and gentle orography (Figure 1).

In other regions of Europe, in Neolithic and Copper Age contexts, sheep farming seems to have focused on meat production [91]. The same occurred at different sites of the Iberian Peninsula such as Mercador (Mouräo, Portugal), Penedo do Lexim (Mafra, Portugal), and La Minilla (La Rambla, Córdoba) where the mortality rate of individuals younger than 24 months might indicate a meat-based use [8,52].

However, in the cases of Cabezo Jure (Alosno, Huelva), Ciudad de la Justicia (Jaén), IA Corte Inglés (Jaén), Les Moreres (Crevillent, Alicante), Monte da Tumba (Torräo, Portugal), and Perdigões (Reguengos de Monsaraz, Portugal), the slaughter or death of the sheep predominantly occurred during juvenile or adult stages [43,48,54,64], which suggests a comprehensive exploitation of livestock, not only for meat, but also for secondary resources such as wool, milk, hides, manure, etc. Lastly, data from the mid-3rd millennium BC at Los Castillejos de Montefrío (Montefrío, Granada), Marinaleda (Sevilla), and Ota (Alenquer, Portugal) reveal a high number of adult individuals, which could indicate a trend towards the exploitation of these secondary products. Faunal studies of Late Neolithic–Copper Age settlements with the most identified and individualised sheep remains show the difficulty in differentiating between Ovis/Capra in infant and juvenile specimens. At Valencina de la Concepción (Sevilla), the age of death or sacrifice is assessed based on the ovicaprid group, with one-fifth of the animals being slaughtered before the age of nine months, and slightly less than half being older than two years [42]. The same was found at Zambujal, where a joint assessment of the age of ovicaprids estimated 180 individuals under two years of age and 194 individuals older than two years [40].

The presence of loom weights, crescent-shaped objects, and “cheese strainers” in archaeological contexts could also be associated with the exploitation of secondary products (wool and milk), whereas their absence might indicate otherwise. One clear example of this is the settlement of Les Moreres (Crevillent, Alicante) where a large number of crescents and loom weights were recovered from two huts dating to the final centuries of the Copper Age [92], which are related to textile and weaving production. The presence of *Ovis aries* was confirmed in this context [55]. Conversely, at Mercador, the absence of these artefacts suggests a meat-based exploitation of sheep [52].

However, it is essential to approach these findings with caution, as the earliest known textile discoveries in the Iberian Peninsula were of plant-based fibres. [93]. In some cases, the bias in faunal information that reaches us through the archaeological record should be supplemented with other analytical methods to clarify whether sheep and goat husbandry were being used. A recent study on lipids found inside Neolithic ceramics demonstrates that in the northern and Atlantic regions of the Iberian Peninsula, ovicaprine livestock was already being exploited for both meat and dairy production [94].

The “flint dagger” from Wiepenkathen (Germany), found in an isolated discovery in a peat bog by farmers in 1935 and typologically dated to the Late Neolithic, was encased in a sheath made of sheep leather, and decorated with a vegetal pattern. The straps wrapping the artefact were made of cattle hide, the wooden handle was covered with textile fibres from sheep, horse, cow, and goat, and the warp threads were made of plant fibres. This represents the oldest evidence of the use of wool in Europe [95]. However, there has always been an ongoing debate among scholars about the role of sheep as wool producers [60,96], with a more conservative view suggesting the probable absence of woolly sheep breeds in Western Europe before the 3rd millennium BCE [9,97,98]. In fact, the earliest evidence of woollen textiles in Southern Iberia is associated with an early Bronze Age burial [99].

The secondary resources of ovicaprids are principally milk and wool. In ovicaprid husbandry, it has historically been shown that goats are more productive in terms of milk yield [100]. Non-improved sheep breeds provide milk to their offspring for approximately 135 days, with a daily milk yield ranging between 0.21 and 0.33 litres per animal [100]. In this context, goats produce more milk than sheep, in some cases doubling the annual milk production in kilogrammes [101]. Ewes start producing milk between 12 and 18 months after their first lambing, with the age of highest production being between 3 and 5 years [102]. On the other hand, sheep start to produce wool from 12 months, but it is between 2 and 4 years of age that they are most productive. Normally, it is from the age of 6 years that ewes start to produce less, and poorer quality, wool [103]. Therefore, the prevalence of sheep in these herds could be due, in addition to their greater docility, to the exploitation of wool, rather than for dairy production, which is higher in goats.

Several studies have attempted to describe the domestication processes of sheep and the origins of current breeds through genomic analyses [104,105,106]. However, only a few works analyse ancient DNA (aDNA) from bone remains, and all of them employed only mitochondrial approaches [90,107,108]. Nevertheless, recently, paleogenomic techniques were used to infer the domestication process on a *Caprinae* subfamily from Neolithic [109]. In fact, to our knowledge, this is the first study to employ genomic approaches in ancient DNA focused on production uses during the late fourth and third millennia BC.

Currently, the Spanish Merino breed is considered the origin of all contemporary Merino breeds and their derivatives [110]. The oldest written reference to sheep from Southern Iberia comes from Columella (42 A.D.), in his work *De Re Rustica* [2]. Here, he describes the crossbreeding practises utilised by his uncle Marcus to obtain higher-quality wool, noting that white-wool is preferable for its ease of dyeing. Additionally, he highlights the value of the dark and blackish fleece of sheep in Italy (Pollentia) and in Baetica (Corduba). In this study, we go back to the late fourth and third millennia BC to investigate the native sheep of the Iberian Peninsula, which later would serve as the basis used by the Romans to establish the genetic stock that would later give rise to the Spanish Merino sheep [110]. Genomic assays showed how individuals from the Marinaleda breed were highly heterogeneous both in terms of multi-locus heterozigosity and inbreeding coefficients, reflecting individuals with different genetic variants (Figure 2a,b). In fact, three of the samples had similar values, which could indicate that they may belong to the same herd. Individuals from La Minilla reflected this trend more clearly and explicitly in its two individuals, which, according to the inbreeding coefficient, could be related in some degree.

The young males kill-off model is one of the strongest arguments used to support domestication events, and was observed in several Neolithic sites in Southwestern Asia [111]. The Chalcolithic context of the present study situates it in a time of management events of already domesticated livestock. Thus, one key element that supports the concept of a managed herd structure is the finding that 100% of the samples analysed were female. This observation aligns with the management practises that have been in place from historical periods to the present, which typically maintain a high female-to-male ratio, estimated at approximately 1 male for every 30 to 50 females [112].

Moreover, it is important to note that the samples used in the genomic analysis comprised adult animals rather than juveniles, where one might expect to observe a female-to-male ratio closer to 1. This discrepancy further emphasises the implications of management strategies on population dynamics and genetic diversity. However, in the prehistoric communities we have studied, males held a prominently symbolic role, as evidenced by the deposits of butchered crania found at some sites in Southern Iberia [6,61].

The aDNA samples from the studied sheep are grouped by their genomic homology based on the site of origin (Figure 3 and Figure 4a). The differences observed may be attributed to a temporal separation of as much as 941 years between the earliest and latest dates at the various sites, according to radiocarbon dating (Table 2), which may have led to a substantial accumulation of genomic variants. This could have resulted in sheep we assume proceed from a common genetic base becoming highly differentiated due to breeding processes related to the management practises of each time period and geographic location. However, the archaeological records suggest that, during this period, management practises were conducted in a highly similar way, and that these populations did not accumulate as much genetic change as is seen in modern breeds. Thus, we can assume that the differences found are due to the redistribution of allele frequencies that occurred due to the particularities of management and the specific environmental conditions of each geographical location, as observed in modern herds of the same breed [113].

Although the genetic distances between sites are not very large (approximately between 0.08 and 0.12), it is evident that the most genetically similar animals are those from La Minilla and Marinaleda, while the most genetically distant are those from Grañena Baja and La Minilla (Figure 5). Nevertheless, we have detected a significant level of genetic homology between samples from two different sites. Specifically, an individual from Marinaleda (FOS82) shows genetic similarity with an individual from Grañena Baja (FOS113), as illustrated in the dendrogram of genetic distances (Figure 4a,b).

The fact that all the Copper Age samples have more genetic similarities between them than with some of the modern breeds can be explained by the extensive process of selection that has taken place over the last few centuries in these breeds (Figure 6). In fact, in recent decades, increased professionalisation and new diagnostic techniques mean that selection has developed into genetic improvement. Proof of this is the large number of livestock breeds which have their own breeding programmes. However, when we analyse the samples individually, we can determine, based on genetic distances, the level of homology they may have with the samples of current breeds (Figure 7).

At the Marinaleda site, most of the samples analysed are equidistant from the current breeds, but with greater similarity to the Black Merino, a trend that is also observed in the Grañena Baja site (Figure 7a,c). However, one of the samples (FOS059) showed similar genetic distances to ME and MN. This could indicate that this individual resembles the endemic animals, but with some features of breeds with greater wool aptitude.

At the La Minilla site however the remains are genetically closer to one of the White Merino samples, and very close to the two samples of the Segureña breed (Figure 7b). In other words, these sheep are genetically close to modern breeds with a marked aptitude for meat use. In the case of the Minilla, the animals slaughtered, in general, were young (males and females), which could indicate a management more focused on meat production where secondary products such as milk and wool would not have such importance for obtaining local resources [8].

This is the first work to analyse the aDNA of sheep from the late fourth and third millennia BC in the Iberian Peninsula, in which we suggest the presence of different types of management, both of primary and secondary products. This may provide an important knowledge base for further research on the sheep that are believed to have derived from the first Merinos.

## 5. Conclusions

The ovicaprine livestock during the late fourth and third millennia BC, with some exceptions, would have been composed of a greater number of sheep than goats due to their docility and/or wool production. In our work, we have sequenced nine samples of individuals over two years old from the archaeological sites of Marinaleda, Grañena Baja, and La Minilla for genomic study, all of which were sexed as females. This leads us to believe that the ratio of males-to-females was very similar to that of the current herds. In the case of La Minilla, the slaughtering patterns of young animals indicates that we they were used mainly for meat production, which is also suggested by the genomic analysis by homology with breeds of greater meat or joint meat–wool use. In contrast, at Marinaleda and Grañena Baja, the presence of a greater number of skeletal remains of adult female individuals (over two years of age) is more in line with the exploitation of secondary resources (milk and wool), which is supported by the greater genetic resemblance to the ancestral Merino Negro breed. The hypothesis of wool production at the sites we studied does not contradict the dates accepted for the first appearance of sheep’s wool in Europe [9]. Nevertheless, we consider our work to be an initial approach to the study of ancestral sheep in the south of the Iberian Peninsula. Following the genetic evidence of the present study, we suggest that the Iberian sheep of the late fourth and third millennia BC, which constitute a possible genetic basis for the Merino sheep and its derived breeds, may have been used for wool production since 5000 years ago. The livestock breeding interests of our ancestors in each era clearly has some genomic reflection in the traces of selection in these sheep. We believe that future research is needed to construct a diachronic journey to detect these genomic traces and situate them in their corresponding historical eras.

## Figures and Tables

**Table 1:** Minimum number of *Ovis aries* individuals found at sites in Southern Iberia.

Site	* MNIs	** Dates
Valencina de la Concepción (Sevilla)	114 [42], 4 [47], 8 [49]	III millennium BC
Cabezo Juré (Alosno, Huelva)	34 [4]	2500 Cal BC.
Papa Uvas (Aljaraque, Huelva)	6 [58], 5 [59], 1 [56]	3092–3052 Cal BC
Gilena (Sevilla)	3 [44]	End IV millennium/Beginning III millennium BC
Marinaleda (Sevilla)	5 (this paper)	2346–2138 Cal BC
La Minilla (La Rambla, Córdoba)	9 [8]	2834–2470 Cal BC
Torreparedones (Baena, Córdoba)	2 [50]	3020 Cal BC
Iglesia Antigua de Alcolea (Córdoba)	2 [5]	3200 Cal BC
Grañena Baja (Jaén)	2 [6]	Beginning III millennium BC
IA Corte Inglés (Jaén)	3 (Unpublished)	III millennium BC
Ciudad de la Justicia (Jaén)	10 (Unpublished)	III millennium BC
Polideportivo de Martos (Martos, Jaén)	4 [60,61]	Late IV millennium Cal BC
Los Castillejos de Montefrío (Montefrío, Granada)	24 [38]	2325 Cal BC
Cerro de la Virgen (Galera, Granada)	8 [41]	Mid III millennium BC
Les Moreres (Crevillent, Alicante)	4 [55]	Mid/late III millennium BC
Cueva de los Covachos (Almadén de la Plata, Sevilla)	3 [62]	End IV millennium/Beginning III millennium BC
Zambujal (Torres Vedras)	108 [40]	2500 Cal BC
Penedo do Lexim (Mafra)	5 [52]	2890–2620 Cal BC
Ota (Alenquer, Portugal)	6 [53]	First half of III millennium BC
Perdigões (Reguengos de Monsaraz, Portugal).	3 [48], 2 [54]	III millennium BC
Monte da Tumba (Torrão, Portugal)	33 [43]	First half of III millennium BC
Mercador (Mourão, Portugal)	14 [51]	Mid/late III millennium BC
Alcalar (Portimão, Portugal)	5 [63]	2577–2335 Cal BC

* MNIs: Minimum number of individuals. Corresponding reference in square brackets [ ] and numbered. ** Dates of archaeological sites. Cal BC: Date Before Christ calibrated by Radiocarbon; BC: uncalibrated date Before Christ.

**Table 2:** Radiocarbon dating of archaeological sites.

Sites	Code	BP	SD	Cal BC 95.4%	m	Bone Sample	References
La Minilla	CNA-3151	3996	35	2619–2460	2525	*Cervus elaphus*	[8]
La Minilla	CNA-3153	4034	36	2834–2470	2543	*Sus scrofa*	[8]
La Minilla	CNA-3152	4040	35	2835–2472	2552	*Bos taurus*	[8]
Grañena Baja	Beta-573496	4230	30	2910–2697	2858	Human femur	[6]
Grañena Baja	Beta-573497	4330	30	3021–2891	2943	Human tibia	[6]
Grañena Baja	CNA-3197	4347	35	3082–2895	2967	Human bone	[6]
Grañena Baja	CNA-3194	4351	33	3083–2898	2968	Human femur	[6]
Marinaleda	CIRAM-11738	3805	32	2346–2138	2244	*Ovis aries*	This paper

Code: Identification of samples with radiocarbon dating. BP: Before Present; SD: standard deviation; Cal BC 95.4%: atmospheric curve employed: IntCal20 [70].

**Table 3:** Selection of samples, archaeological contexts, and chronological intervals.

Sample	Site	Anatomical Element	Diagnostic Area Age Determination	Archaeological Context	Dating Contexts Interval Cal BC
FOS_113	Grañena Baja	Mandible	Tooth wear in permanent teeth	D2/EN131	3083–2697
FOS_115	Grañena Baja	Skull	Frontal fusion	EN 25	3083–2697
FOS_114	La Minilla	Radius	Fusion proximal epiphysis	MIN89/Z-1(3)	2835–2460
FOS_102	La Minilla	Humerus	Fusion proximal epiphysis	Z-1(4)	2835–2460
FOS_059	Marinaleda	Tibia	Fusion proximal epiphysis	UE40	2346–2138
FOS_066	Marinaleda	Tibia	Fusion proximal epiphysis	UE44	2346–2138
FOS_069	Marinaleda	Humerus	Fusion proximal epiphysis	UE30	2346–2138
FOS_082	Marinaleda	Humerus	Fusion proximal epiphysis	UE82	2346–2138
FOS_092	Marinaleda	Humerus	Fusion proximal epiphysis	UE73	2346–2138

## Data Availability

All data contained within the article.
